# Growth, stress, and acclimation responses to fluctuating temperatures in field and domesticated populations of *Manduca sexta*

**DOI:** 10.1002/ece3.6991

**Published:** 2020-11-11

**Authors:** Joel G. Kingsolver, M. Elizabeth Moore, Christina A. Hill, Kate E. Augustine

**Affiliations:** ^1^ Department of Biology University of North Carolina Chapel Hill NC USA; ^2^ Manaaki Whenua – Landcare Research Auckland New Zealand

**Keywords:** acclimation, fluctuating temperatures, growth, laboratory adaptation, reaction norms, stress, thermal performance curves

## Abstract

Diurnal fluctuations in temperature are ubiquitous in terrestrial environments, and insects and other ectotherms have evolved to tolerate or acclimate to such fluctuations. Few studies have examined whether ectotherms acclimate to diurnal temperature fluctuations, or how natural and domesticated populations differ in their responses to diurnal fluctuations. We examine how diurnally fluctuating temperatures during development affect growth, acclimation, and stress responses for two populations of *Manduca sexta*: a field population that typically experiences wide variation in mean and fluctuations in temperature, and a laboratory population that has been domesticated in nearly constant temperatures for more than 300 generations. Laboratory experiments showed that diurnal fluctuations throughout larval development reduced pupal mass for the laboratory but not the field population. The differing effects of diurnal fluctuations were greatest at higher mean temperature (30°C): Here diurnal fluctuations reduced pupal mass and increased pupal development time for the laboratory population, but had little effect for the field population. We also evaluated how mean and fluctuations in temperature during early larval development affected growth rate during the final larval instar as a function of test temperature. At an intermediate (25°C) mean temperature, both the laboratory and field population showed a positive acclimation response to diurnal fluctuations, in which subsequent growth rate was significantly higher at most test temperatures. In contrast at higher mean temperature (30°C), diurnal fluctuations significantly reduced subsequent growth rate at most test temperatures for the laboratory population, but not for the field population. These results suggest that during domestication in constant temperatures, the laboratory population has lost the capacity to tolerate or acclimate to high and fluctuating temperatures. Population differences in acclimation capacity in response to temperature fluctuations have not been previously demonstrated, but they may be important for understanding the evolution of reaction norms and performance curves.

## Graphical abstract

We examine how diurnally fluctuating temperatures during development affect growth, acclimation and stress responses for a field population and a domesticated laboratory population of *Manduca sexta*. Our results show that both population show beneficial acclimation responses to intermediate fluctuating temperature, but the laboratory population has lost the capacity to acclimate to high, fluctuating temperatures.

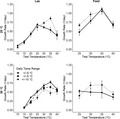

## 1 INTRODUCTION

Temperature and other key environmental factors vary at daily, seasonal and annual time scales, particularly in terrestrial environments. As a result, an ectothermic organism may experience a wide range of environmental and body temperatures during its lifetime, and different life stages or seasonal generations may experience different temperature conditions.

Because temperature strongly influences physiological rate processes (e.g., feeding or metabolic rate), life‐history traits (age or size at maturity), and fitness components (survival or reproduction) in most ectotherms, organisms exhibit a diversity of fixed and plastic responses to variable thermal environments (Cossins & Bowler, 1987; Huey & Bennett, 1990). There are two broad categories of plastic responses to temperature: thermal reaction norms and thermal performance curves (Beaman et al., 2016; Huey & Kingsolver, 1989). A thermal reaction norm represents the phenotypic trait value for some trait of interest as a function of the previous body temperature(s) experienced by the organism or genotype. For example, temperatures experienced throughout development can determine life‐history traits such as final body size and age at maturity. Alternatively, a thermal performance curve (TPC) represents the performance of some rate or trait of an organism—for example, the rate of feeding, metabolism, growth, survival, or reproduction—as a function of its current body temperature..

Responses to current temperatures may also be altered by previous temperatures experienced during development, which we will term time‐dependent effects (Kellermann et al., 2017; Kingsolver et al., 2015). Numerous studies have demonstrated how prior thermal history can reversibly or irreversibly alter subsequent physiological responses to temperature, including heat or cold tolerance, thermal preference, and the thermal sensitivity of metabolic rate (Bowler, 2005). Such time‐dependent effects may have either positive (thermal acclimation) or negative (thermal stress) consequences for performance and fitness (Bowler, 2005, MacLean et al., 2019; Metzger & Schulte, 2017; Zeh et al., 2014).

Many recent studies have emphasized the importance of diurnal fluctuations in temperature for ectotherm performance and fitness in nature (Colinet et al., 2015; Kern et al., 2015). It is important to distinguish three distinct consequences of diurnal variation for organisms. First, because thermal performance curves are nonlinear, mean performance is generally different in constant and fluctuating environments with the same mean temperature. This effect is well‐known and is routinely incorporated into physiological and ecological models of mean performance in variable environments, including changing climates (Deutsch et al., 2008; Ruel & Ayres, 1999). Second, diurnal fluctuations can produce extreme temperatures that exceed lower or upper thermal limits, leading to stress, damage, or death. The negative effects of short‐term exposure to extreme temperatures have been widely documented (Cossins & Bowler, 1987). Third, previous exposure to diurnal fluctuating temperatures earlier in development could alter subsequent responses to temperature—that is, positive (acclimation) or negative (stress) time‐dependent effects (Kellermann et al., 2017; Kingsolver et al., 2015). Whereas numerous studies have demonstrated stress or acclimation responses to prior exposure to constant temperatures or to single heat or cold shocks (Cossins & Bowler, 1987; Sinclair & Chown, 2005), few studies have explored the consequences of diurnal fluctuations for such time‐dependent effects (Cavieres et al., 2018; Kern et al., 2015; Kingsolver et al., 2015). For example, a previous study with domesticated *Manduca sexta* showed that diurnal temperature fluctuations during early larval development increased subsequent larval growth rates at high temperatures, relative to larvae reared at constant temperatures (Kingsolver et al., 2015).

Ectotherms can vary widely in their responses to thermal environments in ways that reflect their evolutionary histories (Angilletta, 2009). Population and species differences in thermal reaction norms, thermal limits, and thermal performance curves have been documented in many taxa, and these differences contribute to adaptation to local environmental temperatures (Frazier et al., 2006; Huey & Bennett, 1987; MacLean et al., 2019; Sunday et al., 2011). However, few studies have evaluated evolutionary differences in organismal responses to diurnal fluctuations, in part because thermal means and fluctuations are often confounded in natural environments (Kellermann et al., 2017).

In this study, we explore the effects of mean temperature and diurnal temperature fluctuations on larval growth, stress, and acclimation responses in the tobacco hornworm *Manduca sexta L*. Our experiments address two related questions. First, how do mean temperatures and diurnal fluctuations throughout larval development affect life‐history traits, such as pupal survival, development time, and mass? Second, do mean temperatures and diurnal fluctuations during early larval development cause acclimation or stress responses in thermal performance curves for growth rate later in larval development? We address these questions for two genetically distinct populations of *M. sexta*: a domesticated population that has been maintained in constant (25–26°C) rearing conditions for over 300 generations, and a field population from piedmont North Carolina that shares a common ancestry with the laboratory population dating to the 1960s. By comparing populations that differ in their recent evolutionary experience to fluctuating thermal environments, our experiments test two predictions: (a) diurnal fluctuations will have fewer negative impacts on thermal reaction norms of life‐history traits in the field than the laboratory population; (b) diurnal fluctuations will generate stronger acclimation or weaker stress responses in TPCs for the field than the laboratory population. Our results provide partial support for these predictions, but the effects of fluctuations depend strongly on mean temperatures.

## 2 MATERIALS AND METHODS

### 2.1 Study system

The Tobacco Hornworm, *Manduca sexta*, occurs across northern South America, Central America, and southern North America. The adult hawkmoths are nectar feeders that can be highly dispersive (Madden & Chamberlin, 1945). The herbivorous larvae feed on a variety of hostplants, primarily in the Solanaceae family, and they are an important agricultural pest on domesticated tobacco in the southeastern United States. The larvae of *M. sexta* typically have 5 instars; toward the end of the final instar, larvae stop feeding, wander off the hostplant to burrow in the soil, and pupate below the soil surface.

Because of its rapid growth and development, large body size and successful maintenance on artificial diets, *M. sexta* has been a model system for insect physiology, development, and ecology for more than half a century. The present study uses two *M. sexta* populations. The laboratory population was first established in ~1980, from *M. sexta* originally derived from animals collected in tobacco fields near Raleigh, NC in the 1960s. This colony has been maintained on a standard artificial diet under constant (25–26°C) temperature conditions and a 14L:10D photocycle without input of additional genetic stock since its founding. Thus, our laboratory population has been in domestication under constant temperatures for over 300 generations. Studies with the field population are based on eggs collected from tobacco plants at the Upper and Lower Coastal Plain Research Stations near Greenville NC in 2016–2018, where populations in this region have 2–3 generations per year. Previous studies show that field and domesticated populations of *M. sexta* can differ in a variety of important traits including feeding and growth rates, critical photoperiod, final size, immune responses, hostplant responses, and upper thermal limits (D'Amico et al., 2001, Diamond et al., 2010, Diamond & Kingsolver, 2010, Diamond & Kingsolver, 2011, Kingsolver, 2007, Kingsolver & Nagle, 2007). Therefore, these populations could also differ in their time‐dependent responses to diurnal fluctuations.

Many aspects of the thermal biology of *Manduca* larvae have been explored. Field measurements show that *M. sexta* larvae experience a wide range of environmental and body temperatures within and between seasonal generations (Case y, 1976, Kingsolver et al., 2007, 2012). *M. sexta* larvae do not actively regulate body temperature except to avoid deleteriously high temperatures above ~42°C (Case y, 1976). Relative to other insect herbivores, *M. sexta* larvae are thermal generalists that can feed, grow, develop, and survive over a wide range of environmental and body temperatures. Under constant temperatures, *M. sexta* can successfully complete egg and larval development to pupation at constant temperatures between 18 and 34°C. In short‐term (24–48 hr) feeding trials, they can maintain positive growth rates for temperatures between 10 and 42°C, with maximum growth rates near 35°C (Kingsolver & Woods, 1997); and they can survive 24 hr heat shocks of 42–43°C (Case y, 1977).

Several previous studies with *M. sexta* have explored the effects of diurnal temperature fluctuations, demonstrating that mean larval growth and developmental rates differ between constant and fluctuating conditions with the same mean temperature (Kingsolver et al., 2009, 2015, Stamp, 1994). For example, our experiments with laboratory *M. sexta* show that diurnal temperature fluctuations increase mean growth and development rates at low mean temperatures (20°C), but decrease these rates at high mean temperatures (30°C) (Kingsolver et al., 2015). These analyses indicate that the effects of fluctuations cannot be fully accounted for by the nonlinearity of thermal performance curves for growth and development: Time‐dependent effects such as stress or acclimation also contribute to these responses, especially at intermediate and higher temperatures (Kingsolver et al., 2015). Similarly, at intermediate mean temperatures, diurnal fluctuations during early larval development can increase the optimal temperature and maximal growth rate in laboratory *M. sexta* (Kingsolver et al., 2015). The effects of high and fluctuating temperatures, and the roles of stress and acclimation responses, are largely unknown for field populations of *M. sexta*; these are the primary focus of the experiments reported here.

### 2.2 Experiments

The basic rearing protocol is similar in both experiments described here. Eggs were placed in petri dishes in a humidified environmental chamber at constant 25°C and 14L:10D photocycle until hatching. Newly hatched larvae (5–10/dish) were randomly assigned to experimental treatments (see below) with abundant food, and the food in each dish was changed regularly as needed. After molting into the 3rd instar, each larva was transferred to an individual dish and maintained individually for the rest of the experiment. Laboratory population larvae were fed on a standard artificial diet; larvae from the field population were fed on an artificial diet with the addition of dried tobacco (*Nicotiana tabacum*) leaves (8.2% dry mass) to serve as a feeding stimulus. Previous studies show that larvae from the laboratory population feed and grow very similarly on diets with and without tobacco (Diamond et al., 2010, Kingsolver, 2007).

#### 2.2.1 Experiment 1: Effects of temperature regimes on thermal reaction norms for life‐history traits

This experiment measured the effects of mean temperature (MT) and the diurnally fluctuating temperatures (DFT) throughout larval development on pupal survival, development time, and mass. The experimental design is the same as that used for a previous study with the laboratory population, with two levels of MT (25°C, 30°C) and three levels of DFT (±0°C, ±5°C, ±10°C). For the field population, eggs were collected from tobacco leaves near Greenville, NC in August and September of 2016 and July and August of 2017 and maintained at 25°C in an environmental chamber until hatching. Newly hatched larvae were assigned to experimental treatments and reared as described above. Starting with the 3rd instar, survival, age (day), and mass (Mettler AT and XSE Toledo microbalances) for each larva were recorded at the start of each subsequent instar, at wandering, and at pupation. Sample sizes were *N* = 516 (field population) and *N* = 273 (laboratory population).

Our analyses focus on survival, development time, and mass at pupation. Because the experiments with the laboratory and field populations were done at different times, we analyzed the laboratory and field data separately. All analyses were done using R (version 3.5.0). Pupal development time and pupal mass were modeled using linear models (function lm) with MT and DFT as factors; pupal survival was modeled as a binomial response using generalized linear models (function glm) with a logit link function.

#### 2.2.2 Experiment 2: Effects of temperature regimes on later thermal performance curves for larval growth

This experiment measured the effects of mean temperature (MT) and the diurnally fluctuating temperatures (DFT) during early larval development (1st through 4th instar) on 24h growth rate of 5th instar larvae at different test temperatures. Experiments with the laboratory population were initiated using eggs from our laboratory colony in 2017, and experiments with the field population were initiated using eggs collected from tobacco leaves near Greenville, NC in July‐August of 2017 and 2018. The rearing treatments (for 1st through 4th instar) included two levels of MT (25°C, 30°C) and two levels of DFT (±0°C, ±10°C); a third DFT treatment (±5°C) was added for the laboratory population at the MT of 30°C (see Section 4).

On the first day after molting into the 5th instar, each larva was weighed and randomly assigned to a test temperature. The test temperatures used for the laboratory population were 15°C, 20°C, 25°C, 30°C, 35°C, and 40°C; for logistical reasons, the test temperatures for the field population were 25°C, 30°C, 35°C, and 40°C. Each test temperature was maintained in a different environmental chamber; the chamber used for each test temperature was altered across experimental trials to account for any differences between chambers. Each larva was maintained at its test temperature in an individual petri dish with abundant diet for 24 hr; at the end of test trial, survival, larval mass, and time were recorded. Larval growth rate was defined as ln[*m_f_*/*m_i_*]/*t*, where m_f_ = final mass at the end of the trial, m_i_ was initial mass at the start of the trial, and *t* was the duration of the trial. This represents the relative rate of growth, relative to the initial mass during the trial. Trial duration was set at 24h for all larvae, but for practical reasons, there was some variation in the actual duration between the initial and final measurements for each individual (mean = 23.8h, *SD* = 1.1 h). Small size at the start of the 5th instar is an indicator of poor condition: Small size at this stage frequently results in additional larval instars and reduces survival to wandering and pupation (Kingsolver, 2007). This is particularly true for the field population, which is more poorly adapted to the artificial diet and shows greater variation in growth rate and size (Diamond et al., 2010, Kingsolver, 2007, Kingsolver & Nagle, 2007). To account for this, we excluded larvae that were less than 250 mg (field population) or 600 mg (laboratory population) at the start of the 5th instar from the analyses (Kingsolver, 2007, D'Amico et al., 2001). The different thresholds reflect the large difference in size between final instar larvae and pupae of field compare with laboratory populations (Diamond et al., 2010, Kingsolver, 2007, Kingsolver & Nagle, 2007). Larvae that did not feed or lost weight during the trial were also excluded. The final sample sizes were *N* = 180 (field population) and *N* = 505 (laboratory population).

For logistical reasons, the experiments at MT = 25°C and MT = 30°C were done at different times, and the experiments with laboratory and field populations were done separately. As a result, we analyzed each of these four cases separately. Preliminary analyses suggested that trial duration had no detectable effects, so for simplicity duration was excluded from all models. Using linear models, we modeled ln[m_f_] (mass at the end of the test trial) as the response variable, with rearing DFT, test temperature, and ln[m_i_] (initial mass) as predictors; DFT and test temperature were modeled as factors and ln[m_i_] as a continuous covariate. We included all two‐way interactions except the interaction of m_i_ with DFT (DFT can alter m_i_ and thus confound the two effects) in the model. We are particularly interested in effects of rearing DFT and the interaction of DFT with test temperature, as these indicate stress (negative) or acclimation (positive) effects of rearing temperature on thermal performance.

## 3 RESULTS

### 3.1 Experiment 1: Effects of temperature regimes on thermal reaction norms for life‐history traits

For the field population, both mean temperature (MT) and diurnally fluctuating temperature (DFT) during development significantly affected survival to pupation, but there was no significant interaction between MT and DFT. Mean survival at MT = 30°C was lower than at 25°C, especially with larger diurnal fluctuations. Overall mean survival across all rearing treatments was 0.48, because field larvae are not well‐adapted to artificial diet, even when tobacco is incorporated into the diet (Kingsolver, 2007, Kingsolver et al., 2009).

**Figure 1:**
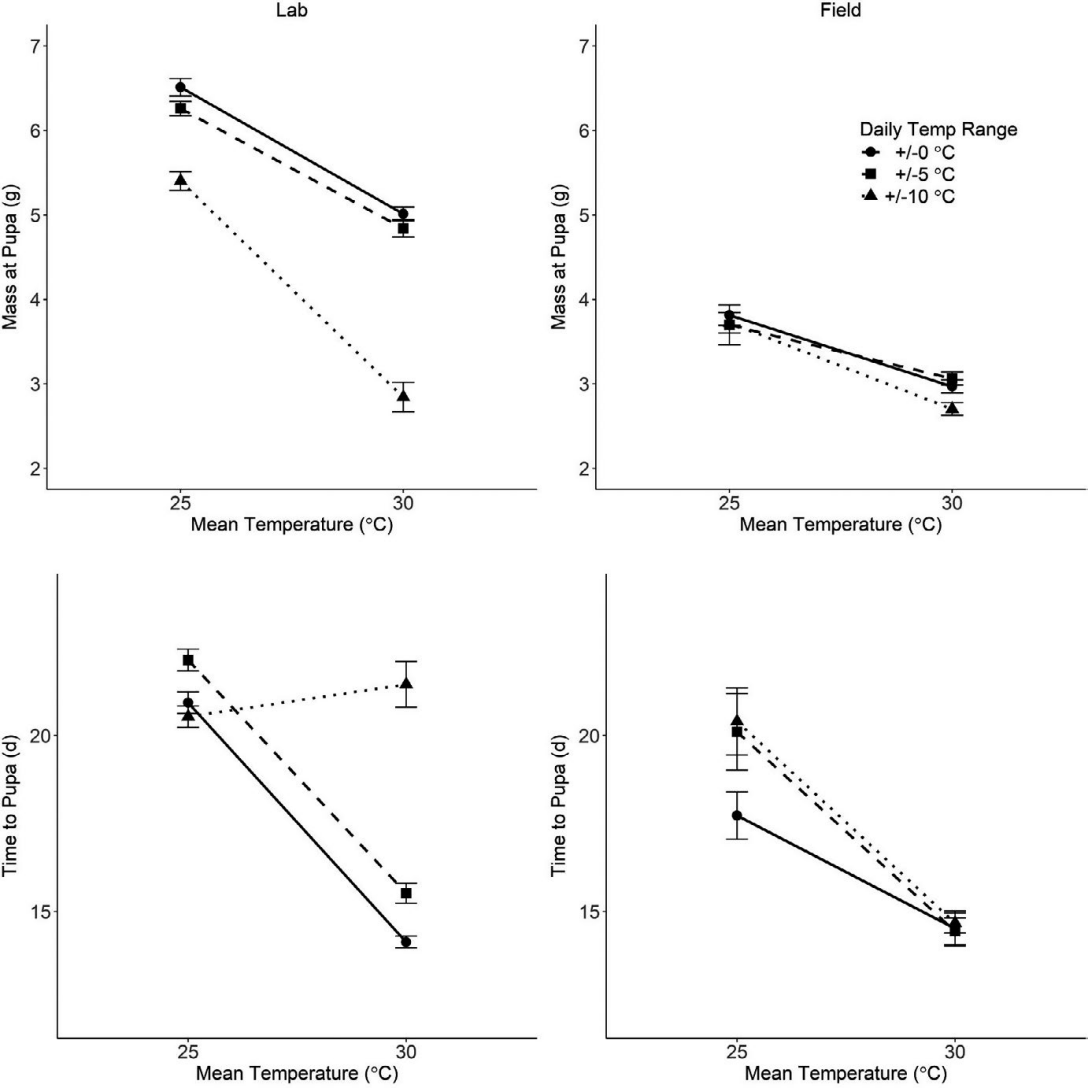
Mean (±1 SE) pupal mass (g, top row) and development time to pupation (d, bottom row) as function of mean temperature during development (°C) for the laboratory population (left column) and field population (right column) of*Manduca sexta*. Line types and symbols in each panel indicate the daily temperature range during development: ±0°C (=constant), solid lines, circles; ±5°C, dashed lines, squares; ±10°C, dotted lines, triangles. Data for the laboratory population (left column) are based on Kingsolver et al. (2015) and are included for comparison

Pupal mass for the field population was significantly affected by MT and DFT, with no significant interaction between MT and DFT (Table 1a). The magnitude of these effects was relatively modest (Figure 1). For example, mean pupal mass was 21% smaller at a mean temperature of 30°C than at 25°C, and 5% smaller in fluctuating (±10°C) than in constant (±0°C) rearing conditions. Development time to pupation was significantly affected by mean temperature but not diurnal fluctuations; there was a marginally significant (*p* = .0504) interaction between MT and DFT (Table 1). Mean development time was shorter at a mean temperature of 30°C than at 25°C; development time was longer in fluctuating than constant conditions at 25°C, but not at a mean temperature of 30°C (Figure 1). These results suggest that for the field population, large diurnal fluctuations at high mean temperature during larval development do not strongly reduce final size or development rate.

**Table 1:** ANOVA tables for Experiment 1: Effects of mean temperature (MT) and diurnal fluctuations in temperature (DFT) on time to pupation and pupal mass

	Df	*F* value	Pr (>F)
A. Field population			
Response: Time to pupation			
MT	1	18.606	<0.001
DFT	1	2.075	0.151
MT:DFT	1	3.867	0.050
Response: Pupal mass			
MT	1	91.038	<0.001
DFT	1	4.259	0.040
MT:DFT	1	0.238	0.626
B. Laboratory population			
Response: Time to pupation			
MT	1	256.739	<0.001
DFT	1	83.579	<0.001
MT:DFT	1	86.138	<0.001
Response: Pupal mass			
MT	1	315.641	<0.001
DFT	1	152.006	<0.001
MT:DFT	1	14.816	<0.001

Note

Analyses for the field (A) and laboratory (B) population are given separately.

Results for the laboratory population have been previously reported and are included here for comparison (Figure 1). There were significant effects of MT, DFT, and their interaction on both pupal mass and development time to pupation. Large (±10°C) diurnal fluctuations reduced mean pupal mass by 20%–40% in the laboratory population, with the largest reductions at higher mean temperature. Similarly, large (±10°C) diurnal fluctuations at higher mean temperature increased mean pupal development time by 50% in the laboratory population (Figure 1). It is striking that under constant temperatures mean development time strongly decreased with increasing mean temperature, but under diurnally fluctuating temperatures mean development time slightly increased with increasing mean temperature. These results suggest that the laboratory population is more sensitive to diurnal temperature fluctuations than the field population, especially at higher mean temperatures.

### 3.2 Experiment 2: Effects of temperature regimes on later thermal performance curves for larval growth

**Figure 2:**
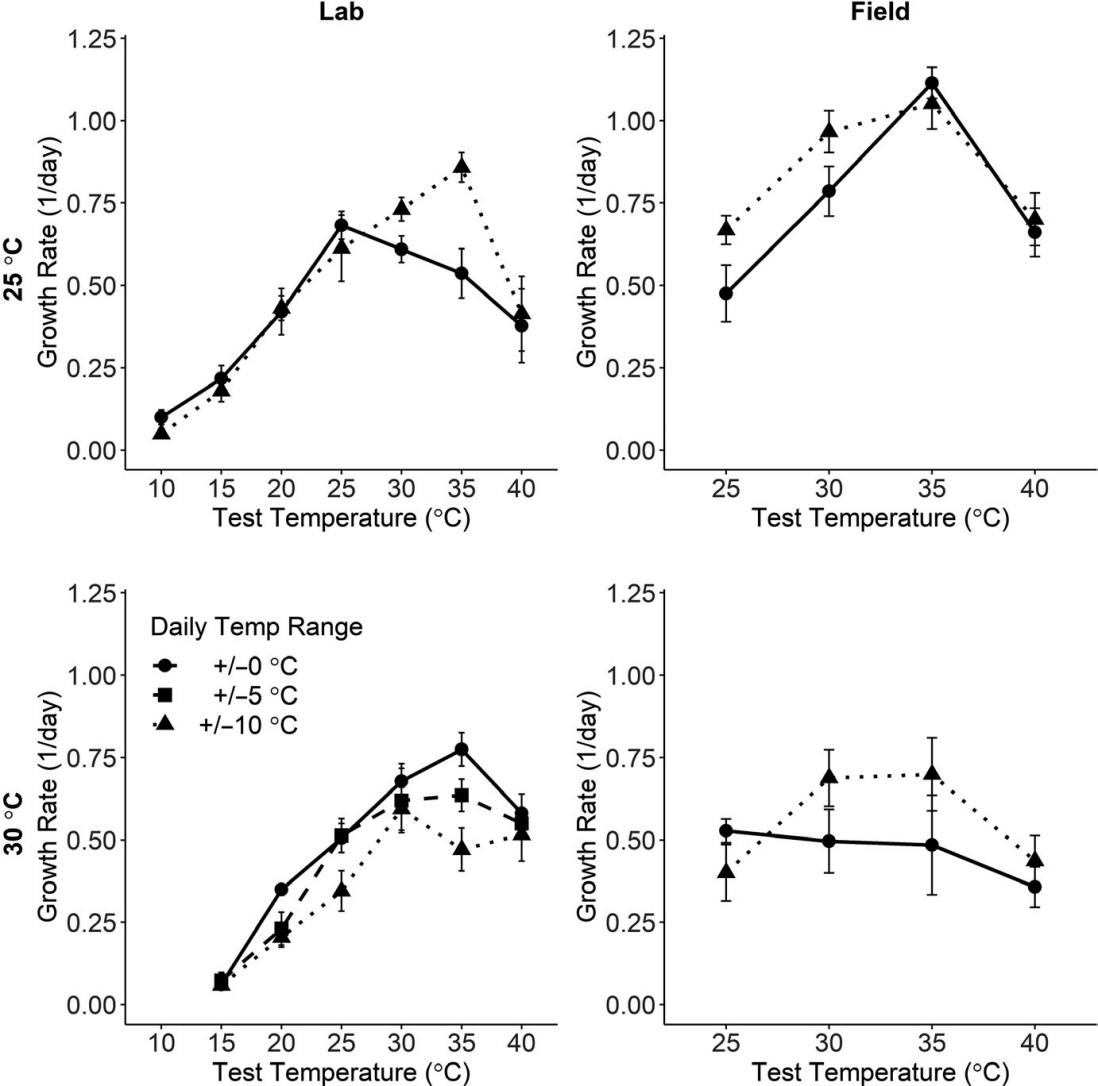
Mean (±1 SE) relative growth rate (day^‐1^) pupal mass of 5th‐instar larvae as a function of test temperature (°C) for the laboratory population (left column) and field population (right column) of*Manduca sexta*, for different larval developmental temperature treatments (from 1st through 4th larval instars). Top row: Mean development temperature = 25°C; bottom row: Mean development temperature = 30°C. Line types and symbols in each panel indicate the daily temperature range during larval development: ±0°C (= constant), solid lines, circles; ±5°C, dashed lines, squares; ±10°C, dotted lines, triangles. Data for the laboratory population at a mean development temperature of 25°C (left top panel) are based on Kingsolver et al. (2015) and are included for comparison

For the field population reared at a mean temperature of 25°C, final mass at the end of the growth trial was significantly affected by test temperature, diurnal fluctuations, and their interaction, indicating that diurnal fluctuations during development affected thermal sensitivity of subsequent larval growth (Table 2). Initial mass and its interaction with test temperature also significantly affected final mass. Mean relative growth rate (day^−1^) was greatest at intermediate temperatures near 35°C (Figure 2). Larvae reared under diurnally fluctuations (±10°C) had higher mean growth rates at most test temperatures than those reared under constant conditions. At a mean temperature of 30°C, final mass for the field population was not significantly affected by diurnal fluctuations or their interaction with test temperature (Table 2). Mean growth rate was higher at intermediate (30–35°C) test temperatures for the fluctuating than constant rearing temperatures (Figure 2), but (because of the larger variability) these differences were not significant (Table 2). These results suggest that diurnal fluctuations during development may have positive (acclimation) effects on larval growth rate for field *M. sexta* at both intermediate and high mean temperatures.

**Table 2:** ANOVA tables for Experiment 2: Effects of test temperature (TT), diurnally fluctuating rearing temperature (DFT), and initial mass (*m_i_*) on final mass (*m_f_*) at the end of the 24 hr test period, for 5th instar *M. sexta* larvae

Effect	Df	*F* value	Pr (>*F*)
A. Field Population			
Mean Temperature = 25°C			
TT	3	29.726	<0.001
DFT	1	17.881	<0.001
*m_i_*	1	5,007.070	<0.001
TT:DFT	3	4.504	0.004
TT: m_i_	3	45.641	<0.001
Mean Temperature = 30°C			
TT	3	6.9999	<0.001
DFT	1	0.0004	0.984
*m_i_*	1	271.790	<0.001
TT:DFT	3	0.606	0.613
TT: *m_i_*	3	4.577	0.005
B. Laboratory Population			
Mean Temperature = 25°C			
TT	6	67.197	<0.001
DFT	1	20.289	<0.001
*m_i_*	1	330.647	<0.001
TT:DFT	6	4.822	<0.001
TT: *m_i_*	6	6.479	<0.001
Mean Temperature = 30°C			
TT	5	54.075	<0.001
DFT	2	118.178	<0.001
*m_i_*	1	542.433	<0.001
TT:DFT	10	3.913	<0.001
TT: *m_i_*	5	4.460	<0.001

Note

Masses (*m_i_* and *m_f_*) were log‐transformed for each analysis to achieve normality. Analyses for the two mean temperatures (MT) of 25°C and 30°C are given separately, because the experiments were done at different times (see text). Analyses for the field (A) and laboratory (B) population are given separately.

For the laboratory population reared at a mean temperature of 30°C, final mass was significantly affected by rearing DFT, test temperature, initial mass, and the interaction between rearing DFT and test temperature (Table 2). Mean growth rate was again greatest at intermediate temperatures of 30–35°C (Figure 2). The laboratory population larvae reared under diurnally fluctuating conditions had lower mean growth rates than those reared under constant conditions for all test temperatures above 15°C, especially for the ± 10°C treatment group (Figure 2). This suggests diurnal fluctuations at high mean temperature during development have negative (stressful) impacts on subsequent larval growth in the laboratory population.

Results for laboratory population at a mean temperature of 25°C have already been described and are included here for comparison. Final mass was significantly affected by rearing DFT, test temperature, initial mass, and the interactions among these predictors (Table 2). Mean growth rate was again greatest at intermediate temperatures of 25–35°C (Figure 2). Laboratory larvae reared under diurnally fluctuating temperatures (±10°C) had similar mean growth rates of those reared under constant conditions for all test temperatures below 25°C, but had higher growth rates at test temperatures of 30–35°C (Figure 2). This result suggests a positive response of laboratory larvae to diurnal fluctuations at this mean temperature, increasing maximal growth rate, and the optimal temperature for growth (Figure 2). Unlike the field population, the laboratory population exhibits positive (acclimation) responses to fluctuations at intermediate mean temperatures, but negative (stress) responses to the same fluctuations at high mean temperatures.

## 4 DISCUSSION

### 4.1 Life‐history responses to mean and fluctuating temperatures

Fluctuating temperatures during development have a wide range of effects on performance and life‐history traits in ectotherms (Colinet et al., 2015; Deutsch et al., 2008; Kern et al., 2015; Ruel & Ayres, 1999; Sinclair et al., 2016). Because rates of growth, development, and other biological processes vary nonlinearly with temperature, the effects of diurnal fluctuations on mean performance can change with mean temperature, especially when diurnal high temperatures exceed the optimal temperature for performance (Colinet et al., 2015; Ruel & Ayres, 1999). For example, diurnal fluctuations can increase mean growth rates at low mean temperatures but decrease them at high mean temperatures, because of the time‐dependent stress effects from repeated exposure to high temperatures (Kingsolver et al., 2015). The consequences of repeated exposure to such stressful, sublethal conditions have now been documented in a number of insect systems (Colinet et al., 2015; Sgro et al., 2016; Xing et al., 2014; Zhang et al., 2015).

The laboratory and field populations of *M. sexta* in our study differ in their growth and developmental responses to fluctuating temperature (Figure 1). For example, diurnal fluctuations strongly reduced mean pupal mass in the laboratory population but had little effect in the field population. The differences are particularly striking at the high mean temperature (30°C), where diurnal fluctuations decreased pupal mass and increased development time for the laboratory but not for the field populations (Figure 1). The field population also shows less plasticity to mean temperature (shallower reaction norm slopes) than the laboratory population for these life‐history traits.

Previous studies have documented substantial evolutionary changes in this and other *M. sexta* populations during laboratory domestication, including increases in larval growth rate and final body size, reduced immune responses, and reduced tolerance to novel hostplants (Diamond & Kingsolver, 2010, Diamond & Kingsolver, 2011, Kingsolver, 2007, Kingsolver & Nagle, 2007, D'Amico et al., 2001). The present results suggest that evolution of the laboratory population under constant temperatures has resulted in lower tolerance and greater sensitivity to high, fluctuating temperatures during development. This is consistent with the finding that larval survival at high, constant temperatures (35°C) is lower in the laboratory than the field population (Kingsolver & Nagle, 2007). In contrast, patterns of HSP gene expression in response to heat shocks are similar in the two populations (Alston et al., 2020).

Given the lack of replicate populations in our study, the evolutionary mechanisms and relative roles of selection and drift in these patterns are unknown. Studies with five strains of *Drosophila melanogaster* showed no changes in mean heat tolerance with domestication over 55 generations (Krebs et al., 2001). Similarly, comparisons between laboratory and field populations for nine *Drosophila* species detected few significant differences in heat or cold tolerance (MacLean et al., 2018). Broader reviews of laboratory adaptation in insects (and some nematodes) have documented significant divergence between field and laboratory populations for a variety of life‐history, morphological and physiological traits (Hoffmann & Ross, 2018; MacLean et al., 2018; Sgro & Partidge, 2000). For example, stress response is generally lower in laboratory than field populations of insects (mostly Diptera), probably as a consequence of relaxed selection (Hoffmann & Ross, 2018). None of these studies directly examined responses to fluctuating rearing temperatures, or aspects of thermal sensitivity beyond cold or heat tolerance. It is useful to note that most of the *Drosophila* studies maintained laboratory lines with relative large population sizes (Krebs et al., 2001; MacLean et al., 2018; Sgro & Partidge, 2000); the domestication history of our (and other) *Manduca* laboratory colonies probably involve substantially smaller effective population sizes, increasing the effects of genetic drift and the accumulation of deleterious mutations relative to the field populations. Estimates of genetic variation in field and domesticated populations of *M. sexta* would be helpful in evaluating this issue.

### 4.2 Stress, acclimation, and selection history

Thermal performance curves provide a useful way to characterize the thermal sensitivity of an individual, genotype or population over a range of body temperatures (Huey & Kingsolver, 1989; Huey & Stevenson, 1979). Many studies have evaluated the potential for thermal acclimation of performance curves, whereby constant temperatures during development or an acclimation period alter subsequent performance (Angilletta, 2009, Beaman et al., 2016; Huey et al., 1999). Beneficial acclimation to constant temperatures has been documented in many ectotherms, especially in aquatic systems (Angilletta, 2009, Seebacher & Grigaltchik, 2014; Seebacher et al., 2014), but few studies have explored acclimation to fluctuating temperatures. For example, a recent study with *D. melanogaster* showed that both mean and diurnal fluctuations in rearing temperature affected thermal sensitivity of adult walking speed (Cavieres et al., 2016). In particular, mean optimal temperature for walking speed increased with diurnal fluctuations at both low and high mean temperatures. By contrast, mean maximum performance increased with diurnal fluctuations at low mean temperature, but decreased with fluctuations at high mean temperatures. This finding suggests that the effects of fluctuations on acclimation and stress responses may depend on mean developmental temperatures (Cavieres et al., 2016; Kingsolver et al., 2015).

Our results for the laboratory population of *M. sexta* support this suggestion (Figure 2). Diurnal fluctuations at lower mean temperature (25°C) during development increased the optimal temperature and maximum performance for larval growth rate, the classic signature of beneficial acclimation. Conversely, fluctuations at higher mean temperature (30°C) decreased growth rate at most test temperatures, confirming that high, fluctuating temperatures during development are stressful for this population (Figure 1; (Kingsolver et al., 2015). As a consequence, plastic responses to diurnal fluctuations during development can generate beneficial acclimation or stress‐induced reductions in performance depending on mean temperature conditions.

Note that diurnal fluctuations had little effect on performance at the lowest or highest test temperatures: The acclimation and stress responses we observed were largely restricted to intermediate temperatures (Figure 2). Recent studies in a variety of ectotherms have suggested that there is limited plasticity in upper thermal limits (e.g., critical thermal maximum temperature, CT_max_) (Hoffmann et al., 2013; Kellermann et al., 2017; Seebacher et al., 2014; Sorensen et al., 2016). For *M. sexta* larvae, diurnal fluctuations during development significantly increase mean CT_max_ at intermediate (25 and 28°C) but not at high (30°C) mean temperatures; mean CT_max_ varied from 44 to 46°C across rearing and heat shock treatments (Kingsolver et al., 2016). Upper thermal limits are frequently well above optimal temperatures: For example in *M. sexta*, CT_max_ (44–46°C) far exceed optimal temperatures for short‐term (~35°C) or long‐term (~30°C) rates of larval growth and development (Kingsolver et al., 2015, 2016; Kingsolver & Nagle, 2007; Kingsolver & Woods, 1997). As the result, the potential for acclimation to high or fluctuating temperature may be quite different for upper thermal limits than for other components of thermal sensitivity.

The field population of *M. sexta* also had a positive acclimatory (i.e., increased mean growth rate) response to diurnal fluctuations at a mean temperature of 25°C, but the laboratory and field population had different responses at the high mean rearing temperature (30°C) (Figure 2). In particular, diurnal fluctuations during rearing significantly reduced subsequent larval growth rates for the laboratory population, but not for the field population. The genetic and physiological bases for these differing responses are unknown, but they are consistent with the hypothesis that, during laboratory domestication at constant temperatures, the laboratory population has lost the capacity to acclimate to high, fluctuating temperatures during larval development.

The field and laboratory populations used in this study also differ their adaptation to larval food: whereas laboratory *M. sexta* perform well on both artificial diet and tobacco (their most common natural host in the Southeast US), field *M. sexta* have slower growth, development, and survival on diet than on tobacco (Diamond et al., 2010). There is also greater variability in growth and development rates in field larvae on diet, and some field larvae have additional instars when reared on diet and other lower quality food resources (Kingsolver, 2007, Diamond & Kingsolver, 2010). Larvae that are below a “critical weight” at the start of the 5th instar are likely to have additional instars, and this differs between field and laboratory populations (D'Amico et al., 2001, Davidowitz et al., 2003); this was the rationale for excluding small larvae from the study (see Section 2). However, the effects of the lower resource quality of diet for field *M.sexta* are unlikely to explain the population differences in thermal tolerance and acclimation capacity at high, fluctuating temperatures that we show here. Models and several empirical studies suggest that reduced nutrient availability will reduce maximal growth rate, optimal temperature and upper thermal limits (Huey & Kingsolver, 2019; Thomas et al., 2012). In contrast, we find that laboratory but not field *M. sexta* suffered reduced growth when reared at high and fluctuating temperatures (Figure 2).

Numerous studies have documented evolutionary reductions in environmental tolerance or in adaptive plasticity as a result of relaxed selection (Lahti et al., 2009; Stoks et al., 2016). Experimental evolution studies in the laboratory can yield either increased or decreased plasticity in the traits that are under selection (Garland & Kelly, 2006). Laboratory domestication typically represents a combination of strong selection on some traits and relaxed selection on others; for example in *M. sexta*, domestication produced strong directional selection for increasing larval growth rate and large final size, and relaxed selection for hostplant defensive chemicals, immune response and heat tolerance (D'Amico et al., 2001; Diamond et al., 2010; Diamond & Kingsolver, 2011; Kingsolver & Nagle, 2007). Studies of laboratory domestication provide a useful tool for understanding how changes in the direction and strength of selection affect the evolution of plasticity (Garland & Kelly, 2006). Population differences in acclimation capacity in response to temperature fluctuations have not been previously demonstrated, but may be important for understanding the evolution of thermal reaction norms and thermal performance curves.

## CONFLICT OF INTEREST

The authors declare that they have no conflicts of interest.

## AUTHOR CONTRIBUTIONS

**Joel G. Kingsolver**: Conceptualization (lead); Formal analysis (lead); Methodology (lead); Project administration (lead); Resources (lead); Visualization (supporting); Writing‐original draft (lead); Writing‐review & editing (lead). **M. Elizabeth Moore**: Formal analysis (supporting); Investigation (equal); Methodology (supporting); Project administration (supporting); Visualization (equal); Writing‐original draft (supporting); Writing‐review & editing (supporting). **Christina A. Hill**: Investigation (equal); Methodology (supporting); Project administration (supporting); Writing‐original draft(supporting); Writing‐review & editing (supporting). **Kate E. Augustine**: Formal analysis (supporting); Investigation (equal); Methodology (supporting); Project administration (supporting); Visualization (equal); Writing‐review & editing (supporting).

## Data Availability

Data presented in the paper are available on Dryad at the time of publication at https://doi.org/10.5061/dryad.15dv41nw3.
